# Refined polysaccharide from *Dendrobium devonianum* resists H1N1 influenza viral infection in mice by activating immunity through the TLR4/MyD88/NF-κB pathway

**DOI:** 10.3389/fimmu.2022.999945

**Published:** 2022-09-13

**Authors:** Xueping Wei, Wei Sun, Pengpeng Zhu, Guoteng Ou, Sheng Zhang, Yuanyuan Li, Jingjin Hu, Xuefeng Qu, Yan Zhong, Wenying Yu, Zhenqiang You, Yin Wang, Yueguo Wu

**Affiliations:** ^1^Institute of Food Science and Engineering, Hangzhou Medical College, Hangzhou, China; ^2^School of Pharmacy, Hangzhou Medical College, Hangzhou, China

**Keywords:** refined polysaccharide from *Dendrobium devonianum* (DVP-1), immune regulation, TLR4/MyD88/NF-κB pathway, H1N1 influenza virus, prevent infection

## Abstract

*Dendrobium* polysaccharide exhibits multiple biological activities, such as immune regulation, antioxidation, and antitumor. However, its resistance to viral infection by stimulating immunity is rarely reported. In this study, we explored the effect and mechanism of DVP-1, a novel polysaccharide from *Dendrobium devonianum*, in the activation of immunity. After being activated by DVP-1, the ability of mice to prevent H1N1 influenza virus infection was investigated. Results of immune regulation showed that DVP-1 significantly improved the immune organ index, lymphocyte proliferation, and mRNA expression level of cytokines, such as IL-1β, IL-4, IL-6, and TNF-α in the spleen. Immunohistochemical results showed that DVP-1 obviously promoted the mucosal immunity in the jejunum tissue. In addition, the expression levels of TLR4, MyD88, and TRAF6 and the phosphorylation levels of TAK1, Erk, JNK, and NF-κB in the spleen were upregulated by DVP-1. The virus infection results showed that the weight loss of mice slowed down, the survival rate increased, the organ index of the lung reduced, and the virus content in the lung decreased after DVP-1 activated immunity. By activating immunity with DVP-1, the production of inflammatory cells and inflammatory factors in BALF, and alveolar as well as peribronchiolar inflammation could be prevented. The results manifested that DVP-1 could resist H1N1 influenza virus infection by activating immunity through the TLR4/MyD88/NF-κB pathway.

## Introduction

Viral pneumonia is an acute respiratory infectious disease caused by virus and is presented by impaired pulmonary gas exchange and parenchymal inflammation of the lungs (1, 2). In recent years, the successive outbreaks of viruses, such as influenza A virus (IAV), avian influenza (H7N9), and novel coronavirus (COVID-19), have emphasized the seriousness of viral pneumonia (3–5). At present, a specific drug to treat viral pneumonia in clinics has not been developed. Some commonly used antiviral drugs, such as ribavirin and interferon, are prone to drug resistance and have many side effects (6). During the COVID-19 epidemic, traditional Chinese medicine (TCM) plays important roles in disease prevention and treatment (7). Moreover, the main method used to treat viral pneumonia with TCM is through regulating the body’s immune system. Increasing number of researchers has begun to focus on the immunomodulatory effects of TCM in recent years because such characteristic is considered to have a unique advantage in the prevention and treatment of viral pneumonia (8, 9). Although TCM has a certain effect on the treatment of viral pneumonia, its mechanism of action has not been fully understood. Among all constituents in TCM, polysaccharides are biomolecules in plants and hold great potential in immune regulation. These biomolecules exhibit antiviral capability and have been attracted considerable attention in recent years (9).

*Dendrobium* is one of the largest genera in flowering plants. This species belongs to the Orchidaceous family with 900–2000 species and is widely distributed in tropical, subtropical, Asian, and northern Australia countries. Approximately 30 *Dendrobium* species are used as traditional medicinal herbs, and 131 compounds have been isolated and possess multiple functions, such as hair growth promotion, neuroprotection, anti-psoriasis, and antioxidant activities (10, 11). Polysaccharides are vital biological macromolecules composed of glucose and mannose monosaccharides and bound by β-1, 4-Manp glycoside. These molecules could be the most abundant components in *Dendrobium. Dendrobium huoshanense*, *Dendrobium officinale*, and *Dendrobium devonianum* contain approximately 20% (12), 35% (13), and 38% (14) polysaccharides, respectively. Polysaccharides are significant bioactive chemical components with antioxidation, immune regulation, hypoglycemic, antiaging, antitumor, liver protection, blood pressure control, lipid lowering, and antifatigue activities (7, 9, 10). However, the efficacy and mechanism of viral infection resistance by immunity regulation of polysaccharides have been rarely reported.

The pharmaceutical activities of polysaccharides have been suggested to be strongly related to their content and composition. In this study, we investigated the antiviral capability of DVP-1, a novel refined polysaccharide from *Dendrobium devonianum* (*D. devonianum*). This polysaccharide is composed of mannose and glucose in the molar ratio of 10.11:1 and has a molecular weight of 9.52×10^4^ Da. Our *in vitro* and *in vivo* experiments indicated that DVP-1 had outstanding immunomodulating effects by directly facilitating the function of macrophages through the activation of the TLR4 and the downstream MAPK and NF-κB signaling pathway. This result indicated the great potential of DVP-1 in antiviral infection by immunoregulation. We also evaluated the effect of DVP-1 on the inhibition of H1N1 influenza viral infection in mice and investigated the underlying mechanism. This study may provide a theoretical basis for the application of TCM typified by *Dendrobium* to prevent virus infection.

## Materials and methods

### Materials and reagents

The stems of *D. devonianum* were purchased from Longling County of Baoshan City, Yunnan Province in Southern China (24°07′ N, 98°25′ E). The voucher specimens were deposited in the Natural Medicine Laboratory of the Institute of Materials Research of the Department of Medicine in the Zhejiang Academy of Medical Sciences in China.

Concanavallin A (Con A), lipopolysaccharide (LPS), and 3-[4,5-dimethylthiazol-2-yl]-2,5-diphenyltetrazolium bromide (MTT) were purchased from Sigma–Aldrich (St. Louis, MO, USA). Antibodies against TLR4, MyD88, TRAF6, TAK1, p-TAK1, GAPDH, IKKα, NF-κB, p-NF-κB, Erk, p-Erk, JNK, p-JNK, c-Jun, and p-c-Jun were obtained from Cell Signaling Technology (Danvers, MA, USA). Horseradish peroxidase-conjugated secondary antibody was supplied by Hangzhou Baoke Biotechnology. Co., Ltd. (Hangzhou, China). Alexa Fluor 488-conjugated AfniPure donkey anti-rabbit IgG was acquired from Jackson Immuno Research Laboratories, Inc. (West Grove, PA, USA). ELISA kit was provided by BD Biosciences (San Jose, CA, USA). Enhanced BCA Protein Assay kit and enhanced chemiluminescence (ECL) kit were obtained from Beyotime Biotechnology (Shanghai, China). Influenza A virus mouse lung-adapted strain A/Puerto Rico/8/1934 (H1N1, PR8) was presented by the First Affiliated Hospital, Zhejiang University School of Medicine.

### Preparation of DVP-1 and crude polysaccharide (CP)

CP and DVP-1 were prepared as we have previously described (15).

### Animal ethics and treatment

The breeding and management of mice conformed with the requirements of the International AAALAC certification. The experimental design was approved by the Ethics Committee of Hangzhou Medical College (Approval Number: 2021–054). All experiments were performed in accordance with relevant guidelines and regulations.

A total of 120 BALB/c male mice (17–19 g, 3–4 weeks old) were bought from Zhejiang Experimental Animal Center (Hangzhou, China). The mice were raised in an SPF-grade barrier system at 20°C–25°C with 50%–60% humidity and 12 h light/12 h dark cycle daily. All mice were free to eat and drink. A mouse model was established as follows. After 4 days of adaptive feeding, 120 mice were randomly distributed into four groups (*n*=30), Control, DVP-1 (60 mg/kg), DVP-1 (15 mg/kg), and CP groups. The mice were orally administered with distilled water, 60 mg/kg DVP-1, 15 mg/kg DVP-1, and 200 mg/kg CP, respectively, for 30 consecutive days. The dosage of CP was referred to the current clinical dose of *Dendrobium* polysaccharide and guaranteed the molecular weight of polysaccharide to be approximately equal to 15 mg/kg DVP-1.

After 30 days, 10 mice were randomly selected to detect immunization function. The remaining 20 mice were infected with 50 μL of influenza A virus mouse lung-adapted strain A/Puerto Rico/8/1934 (H1N1, PR8) virus solution (40 LD_50_/mL) into the left nasal cavity after light anesthetization with isoflurane in each group. Ten infected mice were chosen to investigate the antiinfection ability of DVP-1 on the 6th day, and the remain 10 mice were used to detect the survival status within 14 days after virus infection.

### Determination of organ indices

The mice were anesthetized with 0.1% sodium pentobarbital prior to dissection. The weights of the lungs, spleen, and thymus were measured. Organ indices were calculated as follows: Organ index (mg/g) = organ mass (mg)/animal body mass (g).

### Determination of splenic lymphocytes proliferation

Five mice were randomly selected from each group to remove their spleens. Spleen lymphocyte suspension was prepared in a sterile environment on the 31st day after the oral administration. The suspension was inoculated into a 96-well plate (5×10^6^ cell/mL) and added with ConA (20 μg/mL) or LPS (40 μg/mL). The samples were incubated at 37°C under 5% CO_2_ for 72 h and added with CCK-8. The samples were incubated for another 2 h. Absorbance was detected at the emission wavelength of 450 nm.

### Real-time RT-PCR assay

The total RNA in the spleen, lungs, and intestine homogenates were extracted (Trizol reagent) and reverse transcribed into cDNA after genomic DNA contamination was eliminated using HiScript^®^ Q RT SuperMix for qPCR (+gDNA wiper) (Vazyme Biotech, Nanjing, China) according to the manufacturer’s protocols. About 30 ng of the cDNA was analyzed with ChamQ SYBR qPCR Master Mix (Vazyme Biotech, Nanjing, China) on CFX Touch™ Real-Time PCR Detection System (Bio-Rad, Hercules, USA) according to the manufacturer’s instructions. Relative quantification was performed using 2^−ΔΔCt^ method. The ACT1 gene was used to quantify the mRNA levels of IL-6, IL-1β, IL-4, TNF-α, and PR8. All primers for qRT-PCR were designed using Primer Premier 6.0 software (PREMIER Biosoft International).

### Collection and detection of BALF

BALF was collected as previously mentioned (16). The trachea, which was intubated with a needle when the mouse was euthanized, was completely exposed and lavaged with PBS. BALF was pumped back after the mouse’s chest was pressed. The BALF was stained with DiffQuik. Total cells, macrophages, neutrophils, eosinophils, and lymphocytes were counted under a microscope. According to the manufacturer’s description of the ELISA kit, the BALF was centrifuged at 1500 rpm and 4°C for 5 min. The supernatant was collected and diluted by a factor of 20. The levels of TNF-α, IL-6, IL-1β, and IFN-γ were detected.

### Histopathological examination

The lung tissue, spleen, liver, and jejunum were fixed with 10% formaldehyde solution and embedded in paraffin. The sections were stained with hematoxylin and eosin and observed under an optical microscope.

### Immunohistochemical analysis

The IgA expression in the jejunum tissue was detected by immunohistochemistry according to the method of Miyakawa (17).

### Western blot analysis

The spleen tissue (50 mg) was ground and lysed with 500 μL of radioimmunoprecipitation assay buffer. The obtained cell lysate was centrifuged at 12,000 rpm for 15 min at 4°C. The BCA assay kit was used to detect the protein expression of the lysate. The same amount of protein was separated by SDS-PAGE and transferred onto the PVDF membrane. The membrane was blocked with 5% (w/v) skimmed milk powder prior to the addition of the primary antibody (1:1000). The membrane was incubated overnight at 4°C and then washed with 0.1% Tween-20 in Tris-buffered saline. The secondary antibody was mixed with HRP (1:2000) and incubated at 37°C for 1 h. Protein expression was determined using the ECL kit and the chemiluminescence image detection system (ChemiScope 6000 Exp) and quantitatively analyzed using Image J software.

### Statistical analysis

All data were expressed as mean ± standard error of the mean and analyzed using SPSS 22.0. Statistical comparisons between groups were made *via* one-way ANOVA. P < 0.05 was considered statistically significant.

## Results

### Immunomodulatory effects of DVP-1 in mice

**Figure 1:**
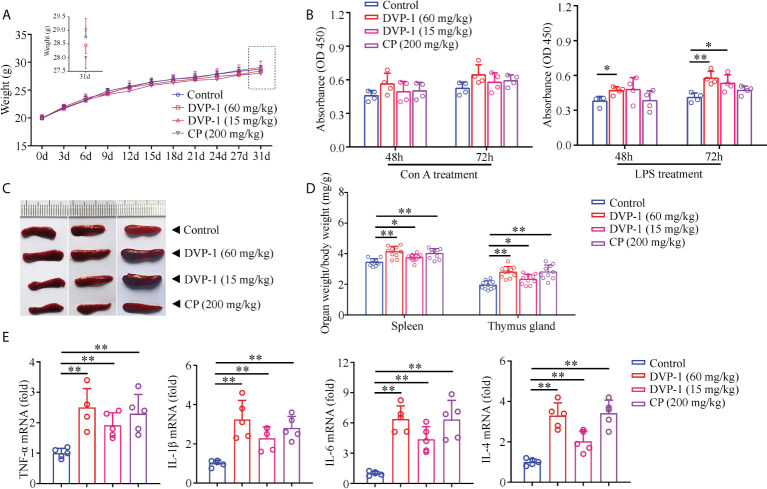
Immunomodulatory effects of DVP-1 *in vivo*. **(A)** Weight changes of mice in each group in 30 days. **(B)** General view of the spleen tissue after anatomy. **(C)** Immune organs index (spleen and thymus). **(D)** Lymphocyte proliferation stimulated by Con A or LPS. **(E)** Level of cytokine mRNA in the spleen using real-time PCR. *p < 0.05, **p < 0.01.

The immunoregulatory effect of DVP-1 was investigated in mice after 30 days of continuous oral administration. After oral administration of DVP-1 and CP for 30 days, the weight of the mice in treatment groups did not change significantly compared with the control group (**Figure 1A**). The results showed that DVP-1 and CP did not significantly affect the lymphoproliferation stimulated by Con A (**Figure 1B**), however, the former could remarkably promote lymphoproliferation under LPS stimulation (**Figure 1B**). In addition, DVP-1 and CP not only increased the organ indices of the spleen and thymus gland but also promoted the mRNA expression levels of TNF-α, IL-1β, IL-6, and IL-4. DVP-1 (60 mg/kg) showed the most significant promoting effect, followed by CP (**Figures 1C–E**). IL-6 exhibited the highest fold changes of up to 6.386-fold and 6.344-fold as stimulated by DVP-1 (60 mg/kg) and CP (200 mg/kg), respectively. IL-4 was upregulated by 3.3-, 3.42-, and 2.02-fold by DVP-1 (60 mg/kg), CP (200 mg/kg), and DVP-1 (15 mg/kg), respectively. The corresponding upregulated fold changes in IL-1β were 3.24-, 2.8-, and 2.28-fold. The corresponding upregulated fold changes in the expression of TNF-α were 2.5-, 2.3-, and 1.92-fold.

### DVP-1 enhancement of the intestinal mucosal immunity in mice

**Figure 2:**
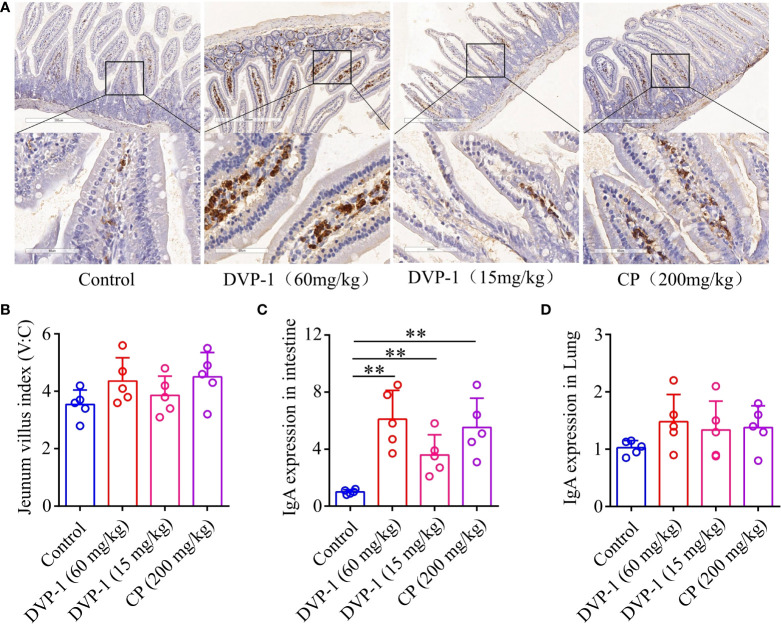
Mucosal immunohistochemistry experiment and real-time PCR. **(A)**, Immunohistochemical detection of IgA expression in jejunum mucosa (8×, 40×). **(B)**, Jejunum villi index. **(C)**, Real-time PCR to detect the expression of IgA in jejunum tissue. **(D)**, Real-time PCR to detect the expression of IgA in lung tissues. ** p < 0.01.

To evaluate the effect of DVP-1 on the mucosal immune system, qRT-PCR and immunohistochemical analysis were performed to measure the IgA mRNA expression and IgA antibody in the jejunum mucosa and lung. DVP-1 outstandingly improved the expression of IgA in the jejunum mucosa of mice (**Figures 2A**), and the result showed that DVP-1 and CP could lightly improve the jejunum villus index. The jejunum villus indices in the control, DVP-1 (60 mg/kg), CP, and DVP-1 (15 mg/kg) were 3.54, 4.36, 4.5, and 3.86, respectively (**Figures 2B**). The qRT-PCR results showed the IgA mRNA expression levels in the intestine were upregulated by 6.1-, 5.52-, and 3.6-fold by DVP-1 (60 mg/kg), CP, and DVP-1 (15 mg/kg), however, there was no significant difference in mRNA expression of IgA in the lung (**Figures 2C, D**).

### DVP-1 activation of the immunity of mice through the TLR4/MyD88/NF-κB signaling pathway

**Figure 3:**
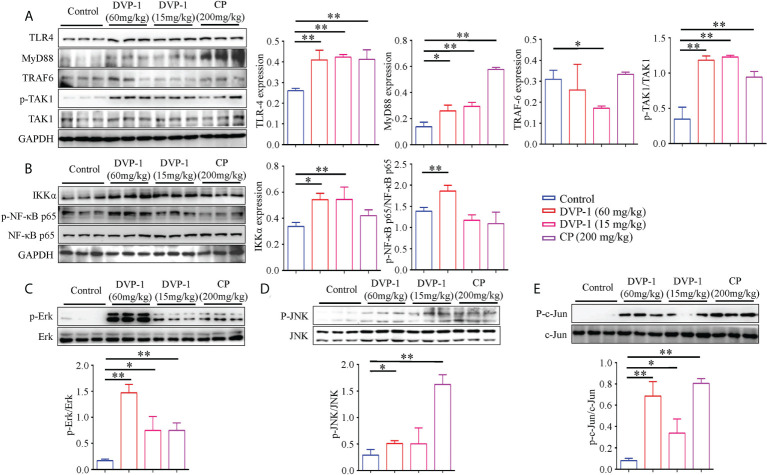
Western blot detection of key proteins in the TLR4/MyD88/NF-κB pathway. **(A)** Protein expression of TLR4, MyD88, TRAF6, TAK1, and phosphorylated TAK1 in spleen tissues as evaluated by Western blot. **(B)** Protein expressions levels of IKKα, and NF-κB and the phosphorylated NF-κB in spleen tissues were evaluated by Western blot. **(C)** Protein expression of total and phosphorylated Erk in spleen tissues as evaluated by Western blot. **(D)** Protein expression of total and phosphorylated JNK in spleen tissues as evaluated by Western blot. **(E)** Protein expression of total and phosphorylated c-Jun in spleen tissues were evaluated by Western blot. Each column represents the mean of three independent experiments. *p < 0.05, **p < 0.01.

To clarify the underlying mechanism of the DVP-1 regulation of immunity, we performed Western blot analysis to reveal the expression and phosphorylation modifications of the key proteins in the TLR4-mediated pathway. Compared with the control group, the expression of TLR4 and MyD88 and the phosphorylation levels of TAK1, Erk, JNK, and NF-κB in the DVP-1 and CP groups were obviously upregulated, while the expression of TRAF6 was significantly downregulated by 0.55-fold in the 15 mg/kg DVP-1 group (**Figures 3A–D**). The expression of TLR4 exhibited similar upregulation effects by nearly 1.6 times in the three treatment groups. Moreover, CP showed the strongest ability of promoting MyD88 expression by 4.2-fold but had no effect on IKKα. Meanwhile, different contents of DVP-1 led to parallel increasing effect on MyD88 and IKKα, which were increased by almost 2-fold and 1.6-fold, respectively. In addition, treatment with 60 mg/kg DVP-1 led to sharper phosphorylation levels of TAK1 and NF-κB by up to 3.4-fold and 1.47-fold (**Figures 3A, B**). CP induced the upregulation of JNK by 5.55-fold, which was the highest among all groups (**Figure 3D**). Among all targets detected, Erk and c-Jun showed the most significant increase in the phosphorylation levels. For Erk, the levels were as high as 8.58-, 4.35-, and 4.35-fold after treatment with 60 mg/kg DVP-1, 15 mg/kg DVP-1, and CP, respectively. The corresponding values for c-Jun were 8.5-, 4.17-, and 9.98-fold (**Figures 3C, E**).

### Anti-infection ability of DVP-1 in mice

**Figure 4:**
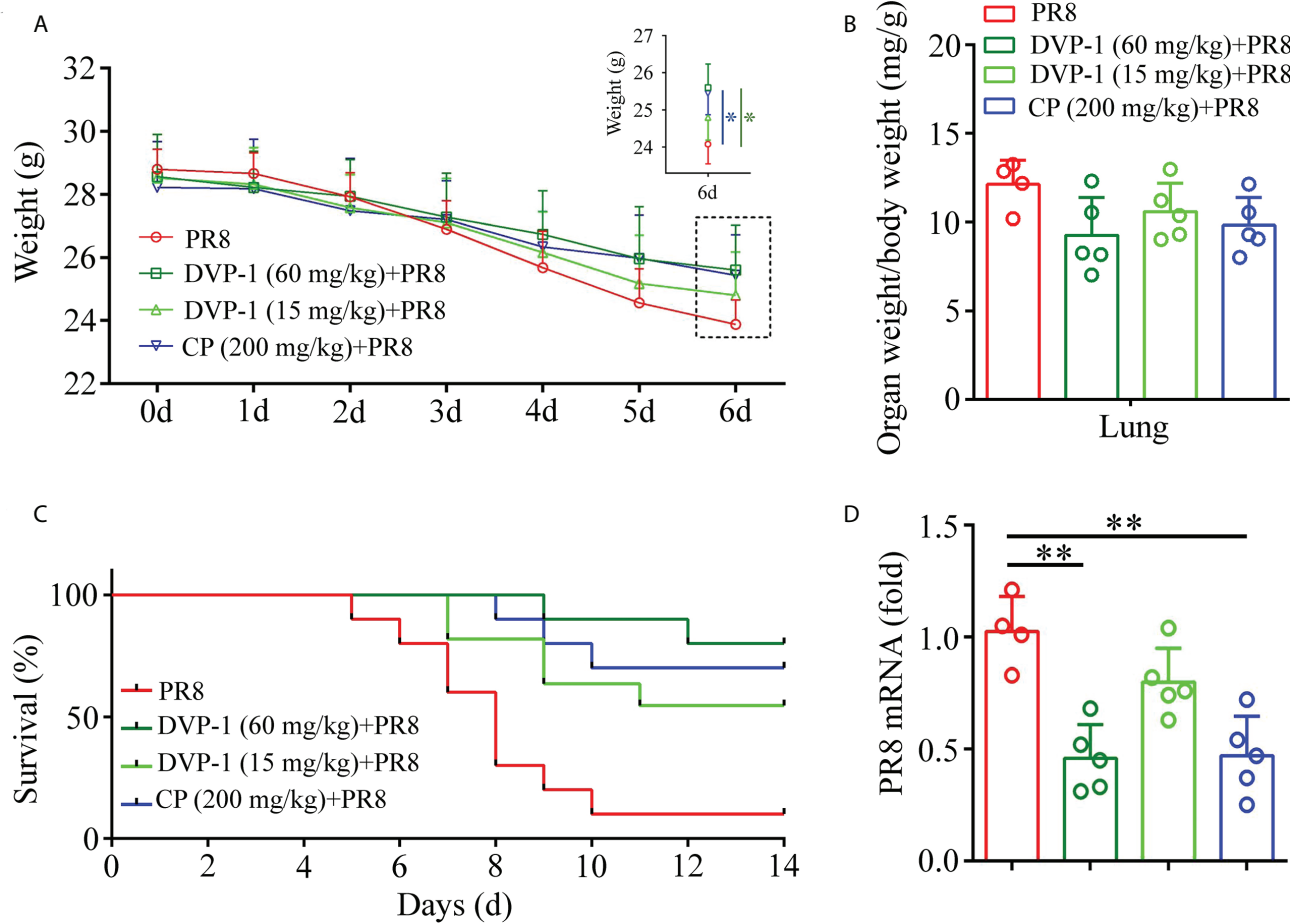
Determination of related indices in mice after virus infection of PR8. **(A)** Body weight change in BALB/c mice. **(B)** Organ index of lung tissue on the 6th day. **(C)** Survival rate of mice within 14 days. **(D)** Virus content in lung tissue on the 6th day. *p < 0.05, **p < 0.01.

After oral administration of DVP-1 and CP for 30 days, mice were infected with PR8 virus strain to explore the antiviral infection ability of DVP-1. The mouse body weight, organ index of lung, viral content, and survival rate were measured on the sixth and fourteenth days after the mouse was infected with PR8 virus strain. Weight results showed that DVP-1 (60 mg/kg) + PR8> CP (200 mg/kg) + PR8> DVP-1 (15 mg/kg) + PR8> PR8 (i.e., 25.6, 25.44, 24.8, and 23.88), similar to the effect in the survival ratio test (**Figures 4A, C**). Only 10% of the mice were still alive after 10 days of PR8 infection. Survival rates of 90%, 70%, and 50% were observed in the DVP-1 (60 mg/kg), CP (200 mg/kg), and DVP-1 (15 mg/kg) groups (**Figure 4C**). The PR8 mRNA expression was reversed, and the PR8 group showed two-fold expression in the DVP-1 (60 mg/kg) and CP (200 mg/kg) groups (**Figure 4D**). No significant effect was found on the organ index of the lung (**Figure 4B**).

### Airway inflammation and cell differentials after PR8 infection

**Figure 5:**
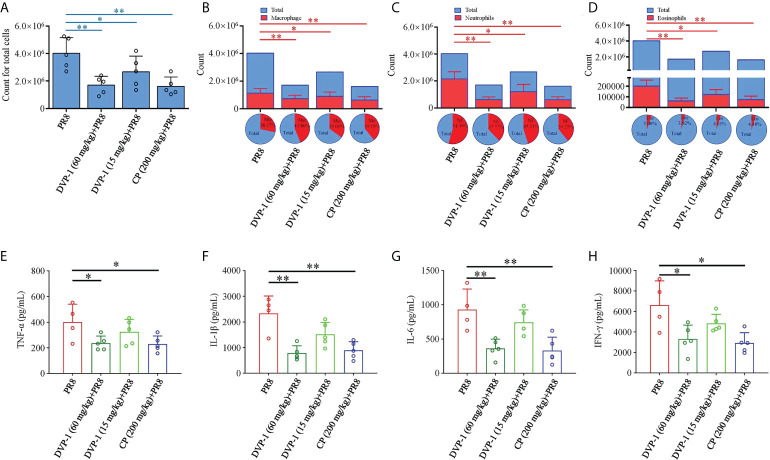
Inflammatory cells and inflammatory factor levels in BALF. **(A–D)**, The number and proportion of total cells **(A)**, macrophages **(B)**, neutrophils **(C)**, and eosinophils **(D)** in BALF on the 6th day after infection of PR8. **(E–H)** The level of inflammatory factors in BALF by ELISA. *p < 0.05, **p < 0.01.

Furthermore, the total and inflammatory cells in the BALF and inflammatory factor levels in the lung were detected to evaluate the level of lung injury in mice. On the 6th day after PR8 infection, the total number of cells, macrophages, neutrophils, and eosinophils in BALF of mice reached a high level. The number of inflammatory cells in BALF of mice, infected with PR8 after immune activation by DVP-1 and CP, was significantly reduced (compared with PR8 group, P<0.05). DVP-1 (60 mg/kg) and CP (200 mg/kg) treatment achieved similar effects. The reduction effect of DVP-1 (15 mg/kg) was weaker than those of the other two treatment groups (**Figures 5A–D**). Further analysis showed that the proportion of macrophages was higher and the proportion of neutrophils was lower in DVP-1 and CP treatment groups than that in PR8 group (**Figures 5A–D**).

The contents of cytokines, namely, TNF-α, IL-1β, IL-6, and IFN-γ, in the different sets had the same trend in the PR8 group, which were almost 1.7-, 3.0-, 3.0-, and 2.0-fold of the results from the DVP-1 (60 mg/kg) and CP (200 mg/kg) groups. Except for the 51% higher content of IL-1β than that of the DVP-1 (15 mg/kg) group, no outstanding difference was observed in the rest of the cytokines (**Figures 5E–H**).

### DVP-1 attenuation of the pulmonary inflammation by PR8

**Figure 6:**
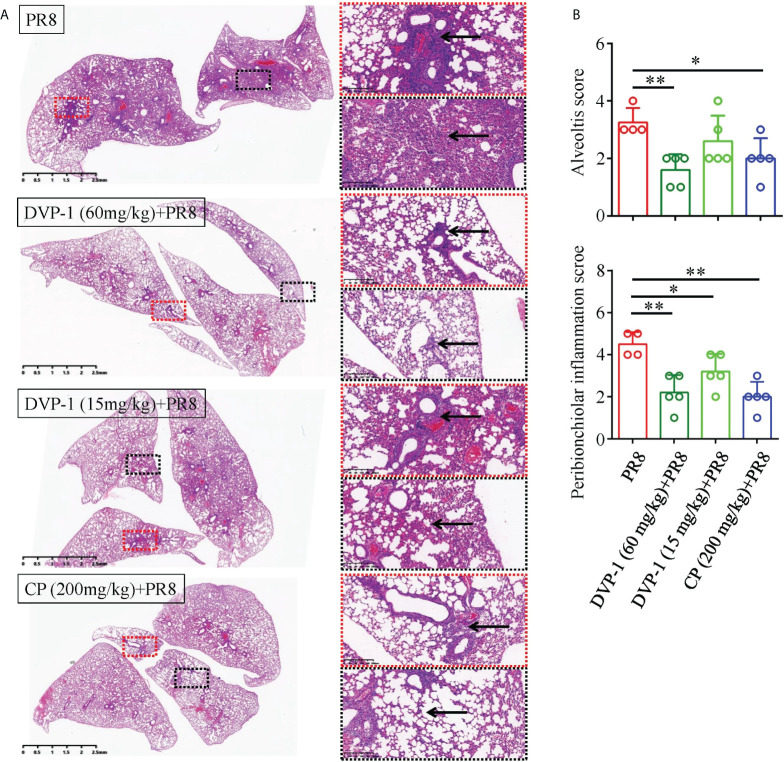
Histopathological results of the lungs. **(A)** Lung tissue was stained by HE to detect histopathological changes (1×, 10×), where the red box represents inflammation around the bronchus, and the black box represents alveolar inflammation. **(B)** Quantitative analysis of alveolar inflammation and peribronchiolar inflammation (inflammation was divided into 1-5 grades from low to high). *p < 0.05, **p < 0.01.

Pathomorphological changes in the lung tissue were observed under an optical microscope to verify the effect of DVP-1 on viral pneumonia in the BALB/c mice. As shown in **Figure 6A**, a large number of inflammatory cells were observed in some alveolar cavities. Obvious inflammatory cells infiltrated the bronchial lumen in the control group, while the alveolar cavity and peribronchiolar inflammation were mild in the three other groups. Moreover, the inflammatory pathological scores of the bronchi and alveoli in the PR8 group reached 3.25 and 4.5, which were twice those of the DVP-1 (60 mg/kg) group (1.6 and 2.2, respectively) (**Figure 6B**).

## Discussion

Natural plant polysaccharides exhibit important biological properties, such as antioxidation, antitumor, antiinflammation, and immunoregulatory activities (18–21). These polysaccharides demonstrate immunomodulatory activity by stimulating lymphocyte proliferation. These biomolecules have been widely perceived as the most promising immunomodulators because of their greatest advantages of relatively nontoxicity and low adverse effects (18, 22). Polysaccharides are abundant in *Dendrobium* (~10%–50%), which is a TCM material that has various biological activities and is widely consumed to prevent chronic diseases by improving health conditions. The multiple functions of *Dendrobium* polysaccharides are associated with immunomodulation. However, the effect of *Dendrobium* polysaccharides on anti-infection through immunoregulation has been rarely investigated. Considering that *Dendrobium* polysaccharides have complex structures and components, we used DVP-1, a refined polysaccharide from *D. devonianum* with clear molecular weight and constituents, to evaluate its performance in protecting mice from PR8 infection (9, 15, 23).

In the immunomodulatory study, the mice were divided into the control, DVP-1 (60 mg/kg), DVP-1 (15 mg/kg), and CP (200 mg/kg) groups. The clinical dose in mice is 200 mg/kg CP, converted from human clinical dose according to body surface area. In addition, 200 mg CP contains about 15 mg of DVP-1. This study aimed to investigate the role of DVP-1 in regulating immunity and clarify whether DVP-1 is the main component of crude polysaccharide in activating immunity. IL-1β, IL-6, IL-4, and TNF-α are important factors involved in immune response. IL-1β has broad immunomodulatory effects and can mediate or directly participate in inflammatory processes. IL-6, also known as a T cell stimulating factor, can stimulate the proliferation of B cells, T cells, and stem cells and promote the production of immunoglobulins by B cells. TNF-α is involved in the development and process of immune regulation (24, 25). Notably, T helper 2 cells (Th2) can regulate humoral immune responses by secreting IL-4, IL-6, and IFN-γ. In addition, TNF-α from T helper 1 cells (Th1) can enhance cellular immunity. IL-6 and TNF-α are mainly produced by tissue macrophages and can trigger a series of innate immune responses (26). In the present study, the secretion of cytokines (i.e., IL-4, IL-6, IL-1β, and TNF-α) in mice was significantly increased in a dose-dependent manner after administration of DVP-1, consistent with previous reports (27–29). The increase in IL-1β, IL-6, IL-4, and TNF-α expression in the spleen indicates the activation of the immune system, resulting in resistance to virus and prevention of virus infection. After oral administration of DVP-1, the ability of lymphocyte proliferation was significantly enhanced. Thus, we speculate that DVP-1 may have better effect of promoting lymphocyte proliferation than CP.

Mucosal immunity is the largest and most complex immunity in the body. The intestinal mucosa is one of its most important components, it consists of secreted IgA and other proteins (30, 31). IgA produced by the plasma cells of the small intestinal lamina propria is the first line of intestinal barrier defense against intestinal toxins and pathogens (32). IgA cannot only prevent antigens from contacting pathogenic microorganisms to maintain an immune homeostasis in the gastrointestinal tract but also affects the composition of intestinal flora and reduce inflammatory responses (33, 34). In our experiment, mucosal immunohistochemical results indicated that DVP-1 and CP could promote the jejunum villi index. The comparison of the expression of IgA in the jejunum and lung tissues showed that DVP-1 and CP had obvious activation effect on intestinal mucosal immunity rather than lung mucosal immunity, the reason may be related to gastrointestinal absorption during the treatment and in-depth research will continue to explore in the future. Besides, Xie et al. (35) found that oral administration of mice with 2.0 g/kg DOP-W3-b, a homogeneous polysaccharide fraction obtained from the stems of *D. officinale*, for 3 or 7 days significantly increased the number of IgA+ cells in the ileum. The regulation of intestinal mucosal immunity by *Dendrobium* polysaccharides may also be related to the effect of the composition and metabolism of intestinal flora (22). Hence, intestinal mucosal immunity could be the mechanism of DVP-1in activating body immunity.

*Dendrobium* polysaccharides can regulate immunity through various pathway, with TLR4/MyD88/NF-κB as the most frequently mentioned pathway. Huang et al. (9) reported that 1, 4-β-D-glucomannan from *D. officinale* activates NF-κB *via* TLR4 to regulate the immune response of human leukemia monocytic cell line (THP-1). Meng et al. (32) proposed that *Dendrobium* polysaccharides activate macrophages to fight pathogens through TLR4, NF-κB, p38 MAPK, Erk, and JNK and can promote the proliferation of macrophages to improve their immune activity. The Erk and NF-κB signaling pathways are possible pathways responsible for the DOP-1 (Polysaccharide from *D. officinale*) regulation of immunity, and researchers believed that *Dendrobium* polysaccharides can directly promote the release of cytokines, such as IL-6, IL-10, and TNF-α, from macrophages to act on target cells and release the reaction products to synthesize NO, these molecules can finally restrain the DNA synthesis of the target cells and remove them (36, 37). We previously showed that DVP-1 can enhance the pinocytosis activity of macrophages *in vitro* by promoting the production of cytokines IL-6, TNF-α, and NO (15). In this study, the Western blot results showed that the expression of TLR4 and MyD88 and the phosphorylation levels of TAK1, Erk, JNK, and NF-κB were obviously upregulated by DVP-1 and CP, consistent with the fructans from *Echinacea* (19). However, CP performed a better effect of increasing MyD88 expression and JNK phosphorylation, but DVP-1 was superior in inducing NF-κB phosphorylation, this difference may be related to the complex composition of polysaccharides in CP. TLR4 is a signaling receptor in the process of many natural polysaccharides entering cells and plays a central role in polysaccharide-induced immune responses and cytokine production (28, 38, 39). TLR4 can stimulate MyD88 to activate TRAF6, a key protein that can promote NF-κB, Erk, Jun, and JNK, thereby regulating interferon production and related cytokines (40, 41). TRAF6 can regulate the downstream expression of genes, such as TAK1 and IKKα, among which TAK1 is a pivotal regulator of immune response signal transduction (42). Based on previous studies, we infer that DVP-1 may activate cells through TLR4/MyD88/NF-κB to regulate gene expression, stimulate cytokine production, and eventually play an immunoregulatory role *in vivo* (9).

Since Reynold proposed that glucan can improve host resistance to viruses, the resistance of polysaccharides from natural plants to viral infection by enhancing immunity has drawn increasing attention (43). Yang et al. (44) reported a polysaccharide (APS-1) from *Angelica sinensis* that can recover the reduction of immune organ index in mice caused by viral infection. Skyberg et al. (45) found that nasal Acai polysaccharides can potentiate innate immunity to protect against pulmonary *Francisella tularensis* and *Burkholderia pseudomallei* infections. Ren et al. (46) proposed that polysaccharide (LNT-1) extracted from *Lentinus edodes* can directly inhibit the replication of the virus in the body and inactivate the virus. Furthermore, plant polysaccharides have been widely proposed to realize such effects *via* regulating the TLR4/MyD88/NF-κB signaling pathway (19, 47). The polysaccharide from *Radix Cyathulae officinalis Kuan* could resist the foot-and-mouth disease viral infection *via* by upregulating DC maturation through the TLR2 and TLR4 signaling pathway and suppressing Treg frequency (48). Astragalus polysaccharide may be employed to prevent the porcine circovirus type 2 infection by decreasing the oxidative stress and the activation of the NF-κB signaling pathway (40). Fructans extracted from *Echinacea* showed extensive antiviral activity against several viruses (including human and avian influenza viruses, H3N2-type IV, and H1N1-type IV) and reversed virus-induced proinflammatory responses by regulating the production of chemokines and cytokines (19). Oxymatrine obtained from sophora root has been proposed to inhibit IAV-induced activations of TLR4, p38 MAPK, and NF-κB pathways, thereby exhibiting anti-IAV and anti-inflammatory activities (49).

Although the resistance of *Dendrobium* polysaccharides to viral infection is rarely reported, we speculate that DVP-1 can prevent viral infection by activating body immunity based on the effective results of the DVP-1 promotion of the expression of key proteins and phosphorylation of the TLR4/MyD88/NF-κB pathway in our previous study and the results from this study. The results affirmed our speculation that DVP-1 not only contributed to the maintenance of the weight of mice but also significantly improved the survival rate and reduced the PR8 content in the lung. These phenomena could be due to the fact that DVP-1 strengthened the organic immunity by stimulating the TLR4/MyD88/NF-κB signaling pathway by which more cytokines, such as IL-1β, IL-4, IL-6, and TNF-α, were generated and released before the viral infection. Ultimately, more PR8 were eliminated by the immune system. This speculation was confirmed by the reduced number of inflammatory cells and levels of several factors, such as TNF-α, IL-6, IL-1β, and IFN-γ, in BALF after PR8 infection as detected in the DVP-1 group. The results of inflammatory cells in BALF also showed that the proportion of macrophages was higher, and the proportion of neutrophils was lower in DVP-1 and CP treatment groups than that in the PR8 group. Macrophages are innate immune cells with well-established roles in the primary response to pathogens, and activated macrophages play a crucial role in nonspecific immune response against tumor and bacterial infection (50). Many plant-derived polysaccharides are known to activate macrophages *in vitro* (51, 52). Our study proved that DVP-1 can activate macrophages and promote the proliferation and differentiation of macrophages through *in vivo* experiments. In addition, better scores of alveolar inflammation and peribronchiolar inflammation from the histopathological results directly proved that the inflammatory response induced by PR8 invasion was alleviated by DVP-1.

The innate immune system is the first line of defense against viral infections and plays a vital role in the early recognition and activation of proinflammatory responses. Viruses invade body and bind to the Toll-like receptors (TLRs), which stimulate the uncontrolled production of proinflammatory interleukins and cytokines that cause inflammatory or cytokine storm. Such phenomenon is commonly accompanied by the rapid increase and accumulation of proinflammatory factors, such as IL-6, IL-8, and IL-1β, which may lead to cell damage. DVP-1 can stimulate the immunity through the TLR4/MyD88/NF-κB pathway and act as a training agent. Such activities result in increased immune response and strengthened killing effect when exposed to viral infections.

Administering DVP-1 in advance before infection can significantly improve the survival rate of mice, reduce the viral content in the body, and improve the inflammation of the alveoli and bronchi of the lungs. In summary, DVP-1 exhibited significant antiviral effects. The polysaccharide could be developed into functional foods and even medicines, especially under the continuous spread of COVID-19 while specific drugs are still lacking.

## Data availability statement

The original contributions presented in the study are included in the article/supplementary material. Further inquiries can be directed to the corresponding authors.

## Ethics statement

The experimental design was approved by the Ethics Committee of Hangzhou Medical College (Approval Number: 2021-054).

## Author contributions

XW: Experimental works, Formal analysis, Writing - original draft. WS: Experimental works, Formal analysis. PZ, GO, XQ, YZ, and WY: Experimental works. SZ: Data analysis, Software. YL: Experimental works. JH: Writing - original draft, Methodology. ZY: Conceptualization, Writing - review & editing. YW: Experimental works, Methodology. YGW: Methodology, Project administration. All authors contributed to the article and approved the submitted version.

## Funding

This work was funded by the Zhejiang Provincial Science and Technology Council (No. LGF21H280008), National Natural Science Foundation of China (No. 82174050), Project of Hangzhou Medical College (No. YS2021015), Huadong Medicine Joint Funds of the Zhejiang Provincial Natural Science Foundation of China (No. LHDMZ22H300009), Zhejiang Provincial Medicinal Health Program (No. 2020PY005 and No. 2021KY130), Project of Educational Commission of Zhejiang Province (No. Y202045371), and Project of Administration of Traditional Chinese Medicine of Zhejiang Province (No. 2022ZB221).

## Conflict of interest

The authors declare that the research was conducted in the absence of any commercial or financial relationships that could be construed as a potential conflict of interest.

## Publisher’s Note

All claims expressed in this article are solely those of the authors and do not necessarily represent those of their affiliated organizations, or those of the publisher, the editors and the reviewers. Any product that may be evaluated in this article, or claim that may be made by its manufacturer, is not guaranteed or endorsed by the publisher.
